# Survey study of changes in total caloric and protein intake across the menopause transition: a preliminary analysis

**DOI:** 10.1080/15502783.2025.2550192

**Published:** 2025-09-01

**Authors:** Valeria V. Silva Ramirez, Anna Oelmann, John Holtje, Landon Shannahan, Gretchen Shelton, Nam Nguyen, Stacy T. Sims, Jacey A. Greece, Laura Pyott, Gabrielle Lyon, Michael J. Ormsbee, Bill I. Campbell

**Affiliations:** a University of South Florida, Performance & Physique Enhancement Lab, Exercise Science & Kinesiology Program, Tampa, FL, USA; b SPRC, Stanford University, Stanford Lifestyle Medicine, Palo Alto, CA, USA; c Department of Research, SPRINZ- AUT, Auckland, New Zealand; d Boston University School of Public Health, Department of Community Health Sciences, Boston, MA, USA; e West Chester University, Department of Mathematics, West Chester, PA, USA; f Department of Muscle-Centric Medicine, Young Medical PC, The Woodlands, TX, USA; g Florida State University, Institute of Sports Sciences & Medicine, Tallahassee, FL, USA; h University of KwaZulu-Natal, Discipline of Biokinetics, Exercise and Leisure Sciences, School of Health Sciences, Durban, South Africa

**Keywords:** Energy intake, muscle mass, menopause, hormones

## Abstract

**Background:**

The menopausal transition is characterized by hormonal shifts, particularly a decline in estrogen, which is associated with increased fat mass and reduced lean body mass. Adequate caloric and protein intake is essential for preserving muscle mass and mitigating these body composition changes. This preliminary analysis examined total energy and protein intake (g/kg body weight) across pre-menopausal, peri-menopausal, and post-menopausal women.

**Methods:**

An anonymous online survey was conducted in resistance-trained females, aged 30–75 years old, using Qualtrics software (Qualtrics, Provo, UT, USA) to document fitness, nutrition, and hormone replacement therapy (HRT) practices during the menopause transition. This preliminary analysis focused on two nutrition-related close-ended questions: “How many calories do you consume (on average) each day?” and “How many grams of protein do you consume (on average) each day?.” Relative protein intake was reported in grams per kilogram of body weight (g/kg). Data is reported with descriptive statistics to estimate mean calorie and relative protein intake per menopausal group and analyzed via one-way ANOVA to compare intake across groups.

**Results:**

There were 1,719 and 1,854 responses for total calorie and protein intakes, respectively. Participants self-reported their menopausal status, with 13.7% identifying as pre-menopausal, 44.5% as peri-menopausal, and 41.9% as post-menopausal. Average estimated caloric intake was the highest in pre-menopausal women (1861 ± 305.9 kcal), followed by peri-menopausal (1823.8 ± 321.6 kcal), and lowest in post-menopausal women (1741.3 ± 333.2 kcal), who consumed significantly fewer calories than both other groups (*p* < 0.001). No significant difference was found between pre-menopausal and peri-menopausal women (*p* = 0.228). Average relative protein intake followed a similar trend: highest in pre-menopausal (1.65 ± 0.53 g/kg), then peri-menopausal (1.63 ± 0.48 g/kg), and lowest in post-menopausal women (1.53 ± 0.55 g/kg), who consumed significantly less than both groups (*p* = 0.004 and *p* < 0.001, respectively). Differences between pre-menopausal and peri-menopausal women were not significant (*p* = 0.696).

**Conclusion:**

Post-menopausal women reported to consume significantly fewer calories and less protein relative to their body weight compared to pre-menopausal and peri-menopausal women, who did not differ significantly from each other. While menopausal status appears to be associated with reduced dietary intake, it is important to note that overall, participants reported protein intakes within the recommended range (1.4–2.0 g/kg) for resistance-trained individuals to build or maintain lean mass. In addition to focusing on menopausal status, future research should investigate whether higher protein intakes are necessary to counteract anabolic resistance associated with aging.

